# Comprehensive Analysis of the Immune and Prognostic Implication of MMP14 in Lung Cancer

**DOI:** 10.1155/2021/5917506

**Published:** 2021-11-24

**Authors:** Chun-long Zheng, Qiang Lu, Nian Zhang, Peng-yu Jing, Ji-peng Zhang, Wu-ping Wang, Gui-zhen Li

**Affiliations:** ^1^Department of Thoracic Surgery, Tangdu Hospital, The Air Force Military Medical University, Xi'an, Shaanxi 710038, China; ^2^Department of Anesthesiology, Tangdu Hospital, The Air Force Military Medical University, Xi'an, Shaanxi 710038, China

## Abstract

More and more studies have indicated an association between immune infiltration in lung cancer and clinical outcomes. Matrix metalloproteinase 14 (MMP14) has been reported to be dysregulated in many types of tumors and involved in the development and progression of tumors. However, its contribution to cancer immunity was rarely reported. In the study, we found that MMP14 expression was distinctly upregulated in lung cancer specimens compared with nontumor lung specimens. High MMP14 expression predicted a poor prognosis of lung squamous cell carcinoma (LUSC) patients. Increased MMP14 expressions were observed to be positively related to high immune infiltration levels in most of the immune cells. A pathway enrichment analysis of 32 MMP14-associated immunomodulators indicated the involvement of T cell receptor signaling pathway and Toll-like receptor signaling pathway. Based on MMP14-associated immunomodulators, we applied multivariate assays to construct multiple-gene risk prediction signatures. We observed that risk scores were independently associated with overall survival. These data highlighted that MMP14 was involved in tumor immunity, indicating that MMP14 could serve as a novel prognostic biomarker and therapeutic target for lung cancer. Our data suggest that the four genes identified in this study may serve as valuable biomarkers of lung cancer patient outcomes.

## 1. Introduction

In the world, lung cancer is the number one cause for male cancer-related death as well as the number two leading cause for female cancer death [1, 2]. Non-small-cell lung cancer (NSCLC) accounts for >85% of lung cancers [3]. To date, the treatment of NSCLC has been improved with the advancement of combined treatments, including surgical resection and systemic chemotherapy, as well as targeted drugs [4, 5]. However, the long-term survivals in patients with NSCLC are still unsatisfactory. Thus, it is necessary for the identification of novel tumor-related markers associated with histopathological features and prognosis, by which the development of advanced diagnostic and treatment options before NSCLC events begin can be promoted.

Among a small number of patients with NSCLC with BRAF (V600E) mutations, ROS1 rearrangements, ALK rearrangements, and EGFR mutations, the use of novel immunosuppressants, such as tyrosine kinase suppressors, significantly improves survivals [6–9]. With the developments of immune checkpoint inhibitors, PD-1 has been widely used for the treatments of NSCLC patients with advanced tumors and achieved a favorable effect [10]. Novel markers including tumor mutation burden, KRAS mutation status, TP53, tumor-infiltration lymphocytes, and PD-L1 expressions have been demonstrated to exhibit important values of clinical responses in treatments based on immune-related methods [11–13]. Nevertheless, more new and sensitive markers are needed to predict or enhance immunotherapy responses.

Sato and his group firstly reported MMP14 as a transmembrane protein which can promote the potential metastasis of tumor cells via activating pro-MMP2 [14]. In recent years, more and more evidences have demonstrated that MMP14 expressions are distinctly increased in many different tumors, and its function involved in cellular migration, inflammation, and angiogenesis is also demonstrated [15, 16]. Moreover, MMP14 expression seems to be higher in NSCLC tissues than noncancerous mucosa, which suggests that cancer patients with high MMP14 expressions have an unfavorable prognosis than those with low MMP14 expressions [17, 18]. Up to now, the mechanism of the oncogenic effect of MMP14 in NSCLC is still unknown. To date, the immunological significance of MMP14 in cancer has rarely been reported. In this study, the status of lymphocytes was systematically evaluated and the connection between MMP14 and NSCLC immunity was clarified, and then, the signaling pathways of MMP14-mediated immune response were regulated.

## 2. Materials and Methods

### 2.1. Raw Data

TCGA datasets (https://portal.gdc.cancer.gov/) with level 3 were used to download transcriptome RNA-SEQ data of 1037 lung cancer cases (nontumor tissues, 108 and cancer tissues, 1037). In addition, the clinical information of all samples was also obtained and concluded.

### 2.2. Evaluation of Immune Infiltration

CIBERSORT is a deconvolution algorithm that characterizes the composition of immune cells in specimens by adopting 547 TAG expressing values [19]. Our group applied the program to assess the relative proportions of 22 infiltrating immune cells in lung cancer's corrected transcriptome information. Then, the corrected transcriptome information was uploaded to the CIBERSORT website. *P* < 0.05 meant that there was a statistical significance.

### 2.3. GO and KEGG Enrichment Analyses

GO and KEGG enrichment analyses of MMP14 coexpressed genes were performed by using clusterProfiler, Enrichment plot, and GGplot2 software packages with the help of R language [20, 21]. It was considered that only *P* and *q* values <0.05 were significantly enriched.

#### 2.3.1. Correlation between MMP14 and Tumor Immune Cell Infiltration

Tumor Immune Estimation Resource was applied to assess the immune infiltrates of dendritic cells, macrophages, neutrophils, CD8+ T cells, CD4+ T cells, and B cells [22]. The site offered several modules, such as Estimation, Correlation, Diff Exp, SCNA, Mutation, Survival, and Gene. Then, the association among immune cell infiltration, survivals, and MMP14 expression was studied.

#### 2.3.2. Immunomodulators

MMP14 related immune regulator from online TISIDB integrated database (http://cis.hku.hk/TISIDB/) in the retrieval, in order to clarify the tumor—the interaction of the immune system [23]. This portal was based on high-throughput screening data. The immunomodulators and immunomodulators which were distinctly connected with MMP14 gene expressions were selected.

#### 2.3.3. Survival Assays

Based on MMP14-related immunomodulators, we developed a prognostic immune-gene signature. In the Cox model, the red pool information criterion was used to select variables step by step. After selecting the immune genes, a prognostic index was generated, called risk score: risk score = *A*_1_ × *X*_1_ + *A*_2_ × *X*_2+−−−−−−−−+_ *A*_*i*_ × *X*_*i*_, where *A*_1_ represents each gene's expression level and *X*_*i*_ represents the risk coefficient of each gene based on the results of the Cox assays. The relationships between immune-related genes and clinical prognosis of lung cancer patients were assessed by adopting Kaplan-Meier assays, log-rank tests, and univariate analysis. A ROC curve was applied for the determination of the prognosis detection of risk scores using the “survivalROC” software package [24].

#### 2.3.4. Statistical Analysis

The distribution of MMP14 expression level in TIMER was shown by box graph, and the differential expression was tested by Wilcoxon test. Kaplan-Meier plot and HR and *P* values or Cox *P* values were used for log-rank tests of survival curve. Spearman correlations were used to determine the correlation between MMP14 expressions and other genes or immune invasion levels in tumor types. Unless otherwise noted, cutoff points are generally set to the median. It was considered that *P* ≤ 0.05 had a statistical significance.

## 3. Results

### 3.1. The Distinct Upregulation of MMP14 and Its Prognostic Value in Lung Cancer

**Figure 1:**
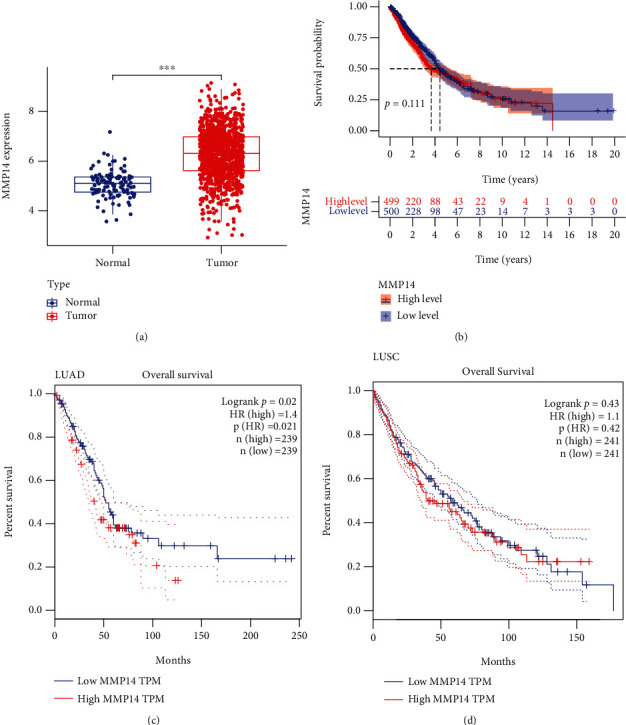
The distinct upregulation of MMP14 and its clinical significance in lung cancer. (a) MMP14 expression was distinctly upregulated in lung cancer specimens from TCGA datasets. (b) Survival assays of 999 patients with lung cancer based on the expression of MMP14. (c, d) Survival assays of patients with (c) LUAD and (d) LUSC based on the expression of MMP14. ^∗∗∗^*P* < 0.001.

To explore the possible function of MMP14 in lung cancer, TCGA datasets were analyzed, and it was found that compared with matched nontumor specimens, MMP14 expression was significantly upregulated in lung cancer specimens (Figure 1(a)). Survival assays showed there was no distinct association between MMP14 dysregulation and survival time of lung cancer patients (Figure 1(b)). However, after we performed subgroup assays, we found that LUAD patients with high MMP14 expressions showed a shorter overall survival than those with low MMP14 expressions (Figure 1(c)), while in LUSC patients, no significant significance was observed (Figure 1(d)).

### 3.2. Distribution of Tumor-Infiltrating Immune Cells (TIICs)

**Figure 2:**
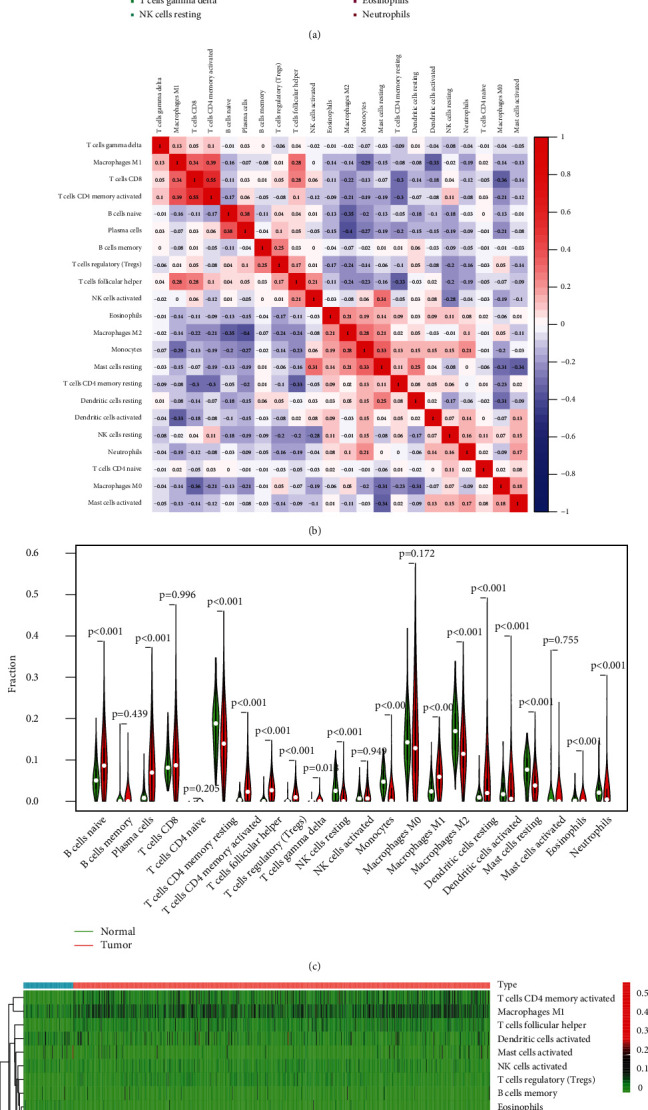
Analysis on the expressions of 22 TIICs and their associations in 1037 lung cancer cases and 108 normal cases. (a) Via analyzing TCGA database, heatmap of 22 TIICs, and immune cells among 1037 lung cancer cases. (b) Pearson correlation coefficient was applied to analyze the matrix of 22 types of TIICs in lung cancer. (c) The dissimilarity in the composition of TIICs between normal specimens and tumor specimens. (d) Heatmaps revealed the change in the immune cell distribution between tumor and normal specimens in lung cancer cohort.

**Figure 3:**
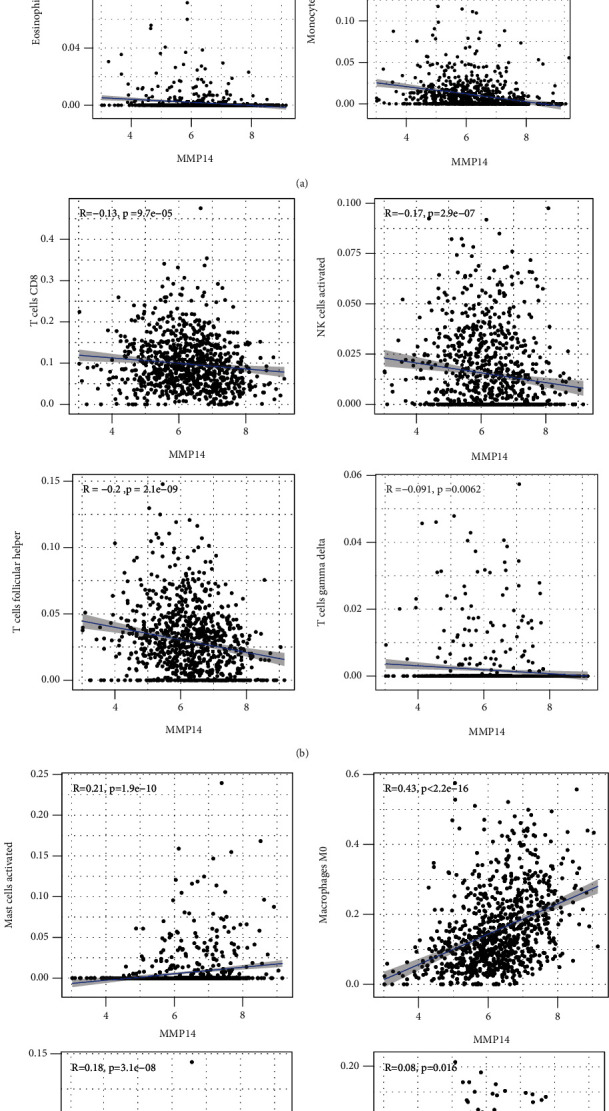
The correlation between MMP14 and immune infiltration level in LUAD. (a, b) The expression of MMP14 was negatively associated with the levels of dendritic cells activated, dendritic cells resting, eosinophils, monocytes, T cell CD8, NK cells activated, T cell follicular helper, and T cell gamma delta. (c) The expression of MMP14 was positively associated with the levels of mast cells activated, macrophage M0, NK cells resting, and macrophage M1.

**Figure 4:**
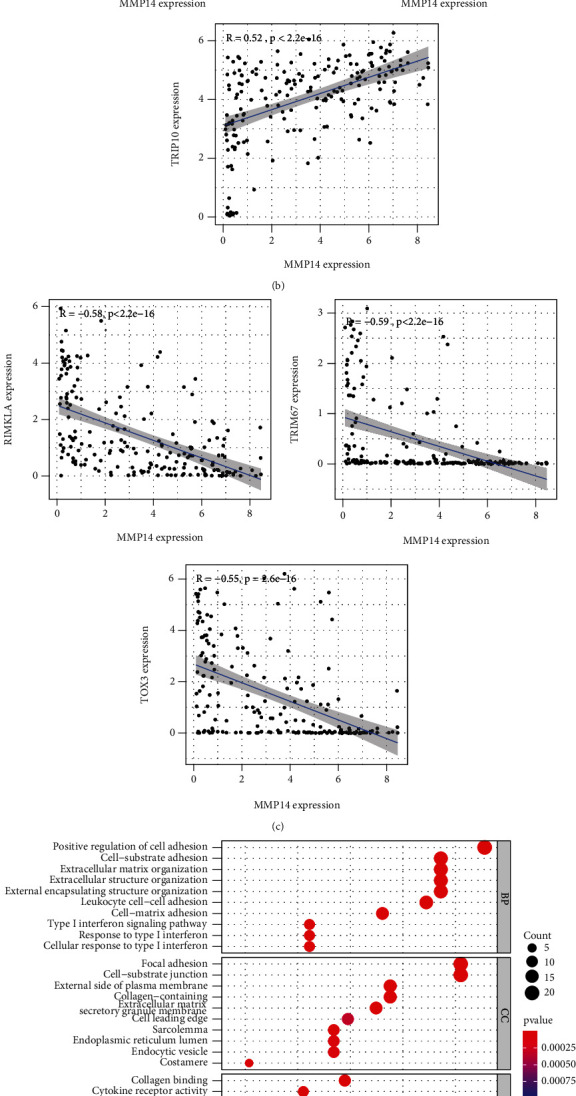
The coexpression genes of MMP14 and GO and KEGG assays. (a–c) The representative coexpression genes of MMP14, such as (a) TNIP1, TMEM43, and TGM2, (b) TNFRSF12A, TIMP1, and TRIP10, and (c) RIMKLA, TRIM67, and TOX3. (d) Gene Ontology (GO) analysis of the coexpression genes of MMP14. (e) KEGG pathway enrichment analysis of the coexpression genes of MMP14.

Figure 2(a) shows the composition of TIICs as well as the correlation between immune cells in lung cancer specimens. We also proved the positive associations between each type of TIICs (Figure 2(b)). In addition, we found significant differences in TIIC composition between normal and lung cancer tissues (Figure 2(c)).  Figure 2(d) shows the different patterns of immune cell infiltration in tumor specimens compared with normal tissues. We observed that eosinophils, dendritic cells resting, dendritic cells activated, monocytes, T cell gamma delta, T cell follicular helper, NK cells activated, and T cell CD8 were positively associated with MMP14 expression (Figures 3(a) and 3(b)). Macrophage M0, NK resting cells, and macrophage M1 were negatively associated with MMP14 expression (Figure 3(c)). Moreover, we analyzed the coexpression genes of MMP14 in TCGA datasets and found a large number of coexpression genes of MMP14. The representative genes are shown in Figures 4(a)–4(c). Gene Ontology contains BPs, CCs, and MFs.  Figure 4(d) shows the top 10. We observed that MMP14-related genes were involved in a lot of BPs, CCs, and MFs, including positive regulation of cell adhesion, cell-substrate adhesion, external encapsulating structure organization, extracellular structure organization, extracellular matrix organization, leukocyte cell-cell adhesion, cell-matrix adhesion, response to type I interferon, cellular response to type I int, type I interferon signaling pathway, focal adhesion, cell-substrate junction, secretory granule membrane, extracellular matrix, collagen-containing, external side of plasma membrane, cell leading edge, sarcolemma, endoplasmic reticulum lumen, endocytic vesicle, costamere, collagen binding, cytokine receptor activity, immune receptor activity, cytokine binding, protease binding, growth factor binding, fibronectin binding, ankyrin binding, MHC class I protein binding, and transforming growth factor beta receptor binding. The top 30 KEGG pathways are displayed in Figure 4(e), including cellular senescence, focal adhesion, proteoglycans in cancer, human T cell leukemia virus 1 infection, shigellosis, hematopoietic cell lineage, regulation of actin cytoskeleton, endocytosis, Epstein-Barr virus infection, ECM-receptor interaction, amoebiasis, FoxO signaling pathway, Hippo signaling pathway, adherens junction, pancreatic cancer, chronic myeloid leukemia, hypertrophic cardiomyopathy, dilated cardiomyopathy, gastric cancer, arrhythmogenic right ventricular cardiomyopathy, antigen processing and presentation, complement and coagulation cascades, colorectal cancer, and TGF-beta signaling pathway.

### 3.3. Association between MMP14 and Immune Cells

**Figure 5:**
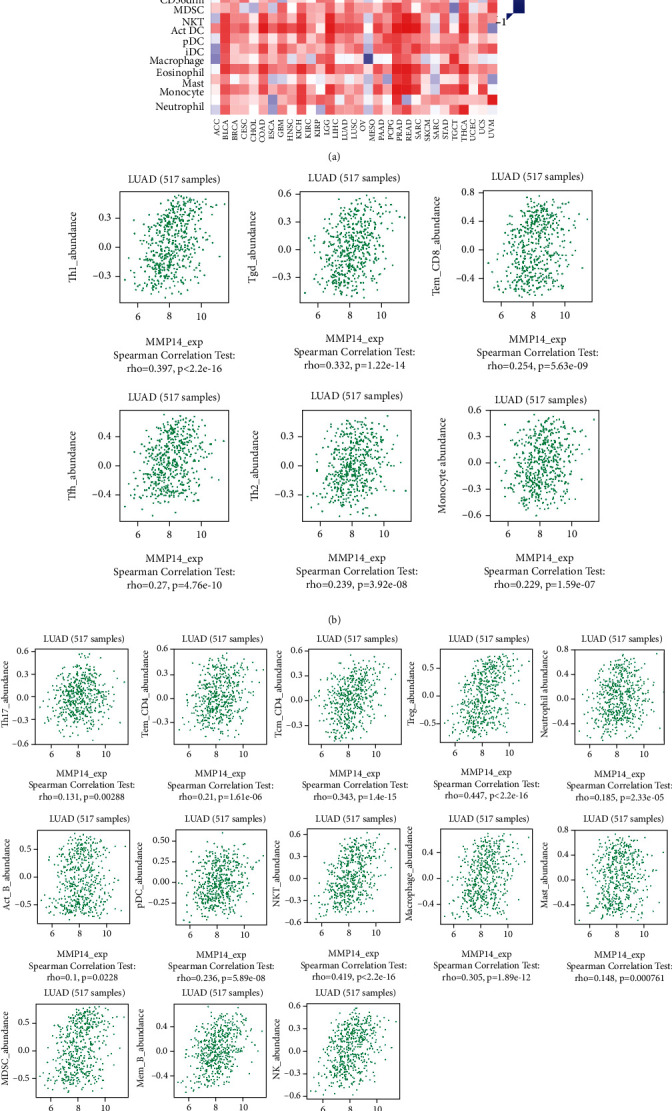
Association between MMP14 expressions and immune cell subsets. (a) Heatmap showed that immune cell types were distinctly correlated with MMP14 expressions in LUAD. (b, c) The associations between MMP14 expressions and immune cells in LUAD.

**Figure 6:**
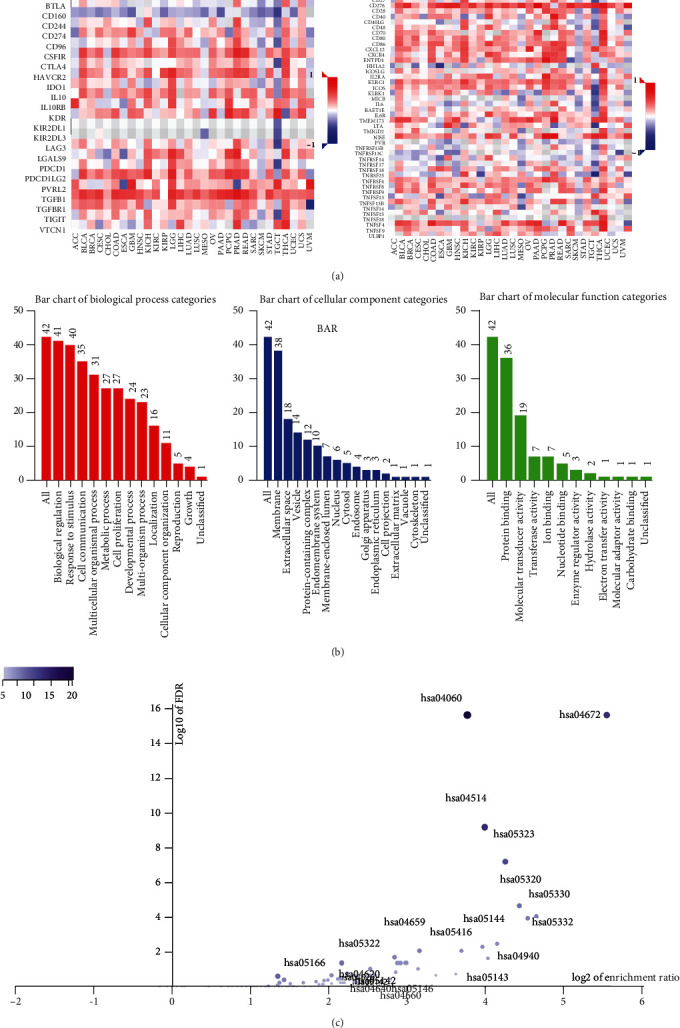
Identification and analysis of immunomodulators associated with MMP14. (a) Heatmap of relationship among immunosuppressants, immunostimulators, and MMP14 gene in LUAD. (b) Gene Ontology annotation of 50 MMP14-associated immunomodulators and 50 closely connected genes in LUAD. (c) KEGG pathway analysis of the abovementioned genes.

We observed the negative or positive associations between several immune subsets and MMP14 mRNA levels (Figures 5(a)–5(c)). Our group also delved into the signaling pathways involved in the function of MMP14 in modulating immune response in lung cancer. 32 immunostimulators (C10orf54, CD80, CD70, CD40, CD28, CD27, CD86, CD276, IL6, IL2RA, ICOSLG, ENTPD1, CXCR4, CXCL12, ICOS, PVR, RAET1E, TMIGD2, IL6R, KLRC1, LTA, MICB, NT5E, TNFRSF9, TNFRSF8, TNFRSF4, TNFRSF13, TNFRSF13B, TNFRSF15, and ULBP1) and 16 immunoinhibitors (VTCN1, TIGIT, TGFB1, PVRL2, PDCD1LG2, PDCD1, LGALS9, LAG3, KDR, IL10RB, IL10, IDO1, HAVCR2, CTLA4, CSF1R, and TGFBR1) that were distinctly correlated with MMP14 in LUAD were identified (Figure 6(a)). Then GO was adopted to annotate the above genes (Figure 6(b)). These genes' KEGG assays indicated that cell adhesion molecules, cytokine-cytokine receptor interaction, malaria, autoimmune thyroid disease, rheumatoid arthritis, Th17 cell differentiation, viral myocarditis, intestinal immune network for IgA production, systemic lupus erythematosus, African trypanosomiasis, graft-versus-host disease, type I diabetes mellitus, amoebiasis, T cell receptor signaling pathway, and Toll-like receptor signaling pathway were related to MMP14-mediated immune events (Figure 6(c)).

### 3.4. Prognostic Developments of MMP14-Related Immunomodulators in Lung Cancer

**Figure 7:**
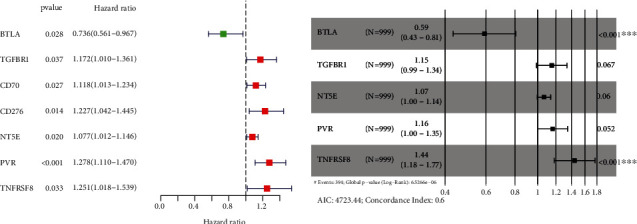
Development of prognostic gene markers based on 48 MMP14-associated immunomodulators and MMP14. (a) Univariate Cox regression analysis of 48 MMP14-associated immunomodulators in TCGA datasets. (b) The HR of genes integrated into prognostic signals is shown in a forest map of lung cancer.

**Figure 8:**
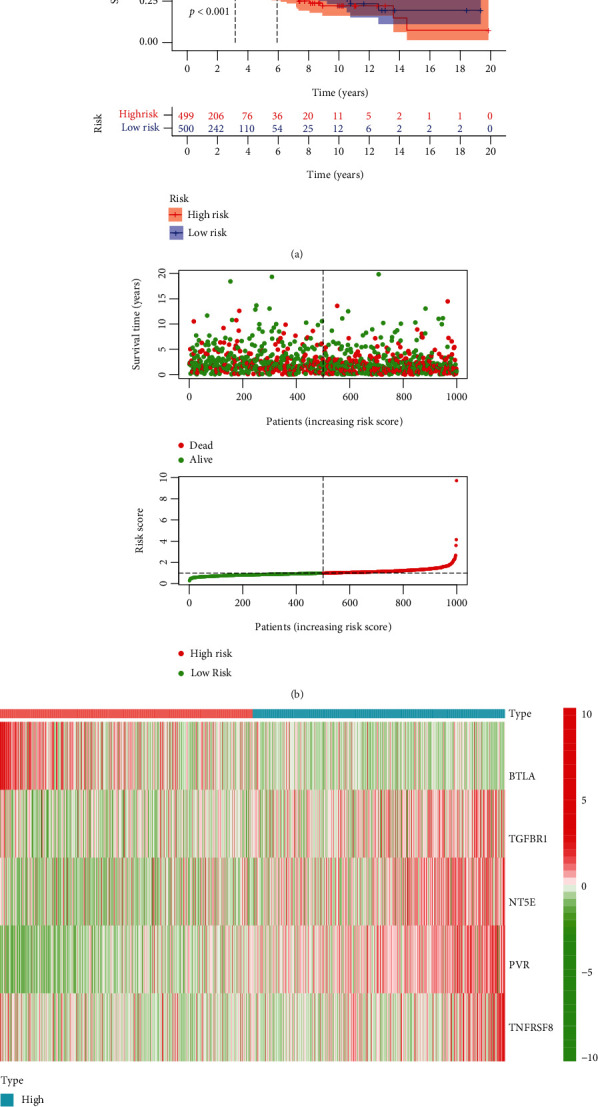
Development of a prognostic immune-related risk signature associated with lung cancer patient outcomes. (a) The overall survival of high- and low-risk lung cancer patients was evaluated using Kaplan-Meier curves. (b, c) Risk score distribution, survival status, and gene expression profile of lung cancer patients.

**Figure 9:**
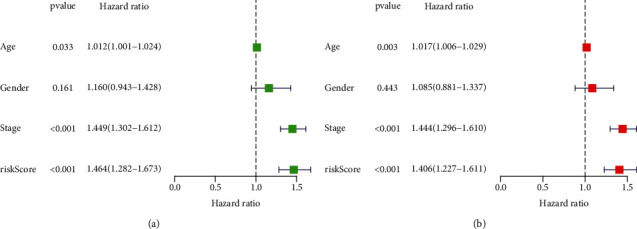
(a) Univariate and (b) multivariate Cox regression analyses of lung cancer risk score and overall survival.

**Figure 10:**
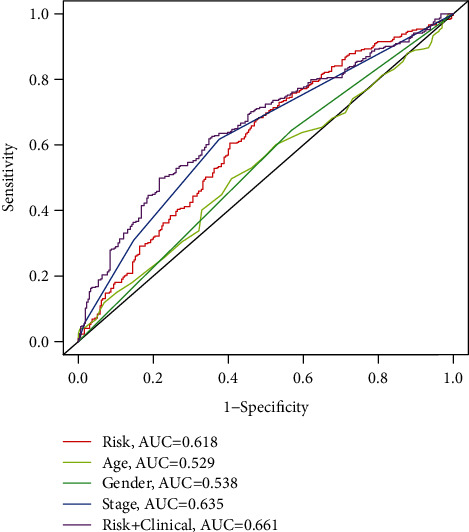
A 5-year time-dependent working characteristic curve in patients with lung cancer. High diagnostic value of risk score was confirmed in the ROC curves depicted in TCGA dataset.

To explore the clinical value of MMP14-related immunomodulators in lung cancer, the above factors were included in univariate assays. Seven prognostic genes were identified, including BTLA, TGFBR1, CD70, CD276, NT5E, PVR, and TNFRSF8 (Figure 7(a)). Further multivariate assays were used to construct a prognostic model. The results are shown in Figure 7(b). After adding the product of each gene's expression value and coefficient, the risk score was calculated. Kaplan-Meier assays showed that patients with high risk exhibited a shorter survival than those with low risk (Figure 8(a)). Figures 8(b) and 8(c) show the distribution of risk scores and survival status as well as characteristic gene expression profiles of LUAD. We further performed univariate assays and found that risk scores were distinctly related to survival (HR = 1.464, 95%CI = 1.282‐1.673, and *P* < 0.001, Figure 9(a)). In addition, multivariate assays showed that risk score (HR = 1.406, 95%CI = 1.227‐1.611, and *P* < 0.001, Figure 9(b)) could independently predict lung cancer prognosis after adjusting for age, sex, and stage. Finally, ROC analysis was carried out, and it was found that the field under the curve (AUC) values of the risk score and stage were 0.618 and 0.635, respectively. When combining risk score with stage, the AUC was 0.661 (Figure 10).

## 4. Discussion

The clinical outcome of NSCLC patients remained unfavorable, and there are large individual differences [25]. Therefore, learning about the complex pathogenesis of NSCLC is critical, so as to delve into the molecular mechanisms involved in the differential outcome of different subtypes of NSCLC [26, 27]. Tumor microenvironment created different kinds of positive environments for cellular proliferation and tumor metastasis, and at the same time, it also played an important role in the induction of tumor cells' drug resistance [28, 29]. Therefore, it is very important to study the influence of NSCLC microenvironment and its potential factors on the clinical processes of NSCLC. At the same time, there is a lot of evidence that some specific genes could be applied to promote cancer diagnosis and personalized treatments.

In this study, we analyzed TCGA datasets and found MMP14 expression was distinctly upregulated in all NSCLC samples compared with nontumor lung specimens, which was consistent with previous several studies. However, there was no significant difference between long-term survivals of patients with high MMP14 expression and those with low MMP14 expression [30, 31]. Then, we performed subgroup assays and found that LUAD patients with a high MMP14 expression level exhibited a shorter overall survival than those with a low MMP14 expression level. However, for LUSC patients, the high MMP14 expression was not correlated with the LUSC patients' clinical outcomes. These findings suggested the more obvious effects of MMP14 on the progression of LUSC than LUAD. More clinical assays were needed to further study the prognostic value of NSCLC patients' MMP14 expression.

First of all, the composition of individual patients' intratumoral immune subsets was evaluated, as several immunotherapies have been exploited to modulate the above cells [32, 33]. For example, T lymphocyte subsets including CD8+ could be regarded as a predictor for the effects of immunotherapies [34, 35]. Based on TCGA datasets, we observed that compared with normal tissues, the composition of 22 immune subsets of NSCLC was significantly altered. This method has been widely used to investigate the role of immune infiltrating cells in various tumors, and these results indicated that the pattern of intraimmunocytoma invasion is associated with NSCLC prognosis. However, until now, it has been very difficult to capture the view of an individual patient infiltrating immune cells in a clinical setting. Therefore, the discovery of novel markers that specified the immune status of patients was very important.

In this study, we provided evidence that MMP14 was correlated with immune cell infiltration in NSCLC. As far as we all know, this study proved that there was a link between MMP14 and tumor immunity, and it was found that MMP14 levels were negatively correlated with the level of activated dendritic cell infiltration, macrophage M1, macrophage M0, eosinophils, dendritic cells resting, NK cells activated, neutrophils, monocytes, mast cells resting, mast cells activated, T cell CD8, T cell follicular helper, and T cell gamma delta. However, the association of MMP14 and immunity should be further demonstrated by other datasets.

Previously, the function of MMP14 has been reported in several types of tumors. For instance, MMP14 empowered tumor-initiating breast cancer cells under hypoxic nutrient-depleted conditions [36]. MMP14 expression was distinctly upregulated in nasopharyngeal carcinoma, and its knockdown suppressed the cell migration and invasion through epithelial-mesenchymal transition [37]. Knockdown of MMP14, regulated by miR-22, was shown to suppress the proliferation and invasion of gastric cancer cells [38]. In lung tumor, MMP14 was observed to enhance the EGFR signaling to promote tumor metastasis and growth [39]. These findings highlighted the oncogenic roles of MMP14 on tumor progression. However, the potential mechanisms remained largely unclear. In this study, we performed gene coexpression networks and performed KEGG assays, finding that the most significant pathways were the cytokine-cytokine receptor interaction, cellular senescence, focal adhesion, proteoglycans in cancer, human papillomavirus infection, human T cell leukemia virus 1 infection, hematopoietic cell lineage, FoxO signaling pathway, and Hippo signaling pathway. Among these pathways, focal adhesion, proteoglycans in cancer, FoxO signaling pathways, and Hippo signaling pathways are distinctly associated with cancers. Besides, the research indicates that focal adhesions are correlated with therapy resistance and play an important part in carcinogenesis and tumor progression as well as metastasis.

We have consistently established immune gene markers for NSCLC by immunomodulators associated with MMP14, and it was found that the risk scores from genetic markers were distinctly related to survivals in NSCLC. The great majority of the immune-related genes incorporated into the prognostic signals are involved in the modulation of T cell activity, which highlighted the importance of T cell-mediated immunity in NSCLC. These data suggested that the risk scores could distinguish between risk populations defined by differential expressions of a set of characteristic genes. Therefore, these results may speed up the developments of signals with good validation for cancer prognosis.

Our research also had certain limitations. Firstly, the study may cause a selection bias due to the retrospective feature. The sample size was insufficient in the validation datasets. Secondly, the expression of MMP14 at protein levels in tumor specimens was not further demonstrated, which was needed to be further demonstrated. Thirdly, the tumor-related function of MMP14 was not explored using in vitro and vivo assays. Further advanced researches were needed to validate our conclusions.

## 5. Conclusion

Our study has suggested that MMP14 might not only be a potential biomarker for poor prognosis in NSCLC, but also play an important part in the microenvironment of MMP14 by regulating tumor invasion of immune cells, indicating that MMP14 can be used as a therapeutic target to modulate antitumor immune responses.

## Data Availability

The datasets used and/or analyzed during the current study are available from the corresponding authors on reasonable request.
